# Draft Genome Sequence of *Salinispora* sp. Strain H7-4, Isolated from Deep-Sea Sediments of the Shikoku Basin

**DOI:** 10.1128/MRA.00834-20

**Published:** 2020-11-05

**Authors:** Dana Ulanova, Yuka Uenaka, Masazumi Sakama, Tetsuya Sakurai

**Affiliations:** a Department of Marine Resource Science, Faculty of Agriculture and Marine Science, Kochi University, Nankoku, Kochi, Japan; b Center for Advanced Marine Core Research, Kochi University, Nankoku, Kochi, Japan; University of Rochester School of Medicine and Dentistry

## Abstract

*Salinispora* spp. are obligate marine actinomycetes reported mainly from shallow tropical and subtropical marine habitats. In this announcement, we present the isolation and draft genome sequence of *Salinispora* sp. strain H7-4 of a new 16S rDNA phylotype from deep-sea sediments of the Shikoku Basin.

## Abstract (precis)

*Salinispora* spp. are obligate marine actinomycetes reported mainly from shallow tropical and subtropical marine habitats. In this announcement, we present the isolation and the draft genome sequence of *Salinispora* sp. strain H7-4 of a new 16S rDNA phylotype from deep-sea sediments of the Shikoku Basin.

## ANNOUNCEMENT

The obligate marine actinomycetes *Salinispora* spp. (*Micromonosporaceae*) are known as a prominent source of bioactive compounds, e.g., salinosporamide A with potent anticancer activity (1). Three described (*Salinispora arenicola, Salinispora pacifica*, and *Salinispora tropica*) and seven proposed *Salinispora* species share 99% 16S rRNA gene sequence identity (2, 3). About 10% of *Salinispora* sp. genomes are dedicated to secondary metabolite biosynthetic gene clusters (BGCs), which often show species-specific and strain-specific distributions (4, 5).

*Salinispora* spp. have been isolated or culture-independently detected from tropical, subtropical, and temperate marine sediments, seaweed, and marine invertebrates, with only a few deep-sea locations reported (6, 7). This study presents the isolation and genome sequencing of a new *Salinispora* sp. strain from deep-sea sediments of the Shikoku Basin.

The surface sediment was collected from the seamount in the Shikoku Basin (30°11.403′N, 136°38.233′E; water depth, 1,712 m) with a grab sampler. The top 2 cm of sediment was scooped and stored at 4°C until processed. The sediment sample was dried in a laminar flow cabinet and directly plated onto AMM, M1 (10× diluted AMM), and Bennet’s agar plates (8, 9), prepared using deep seawater and supplemented with cycloheximide (100 mg liter^−1^). Inoculated plates were cultivated at 28°C for up to 4 months.

**FIG 1:**
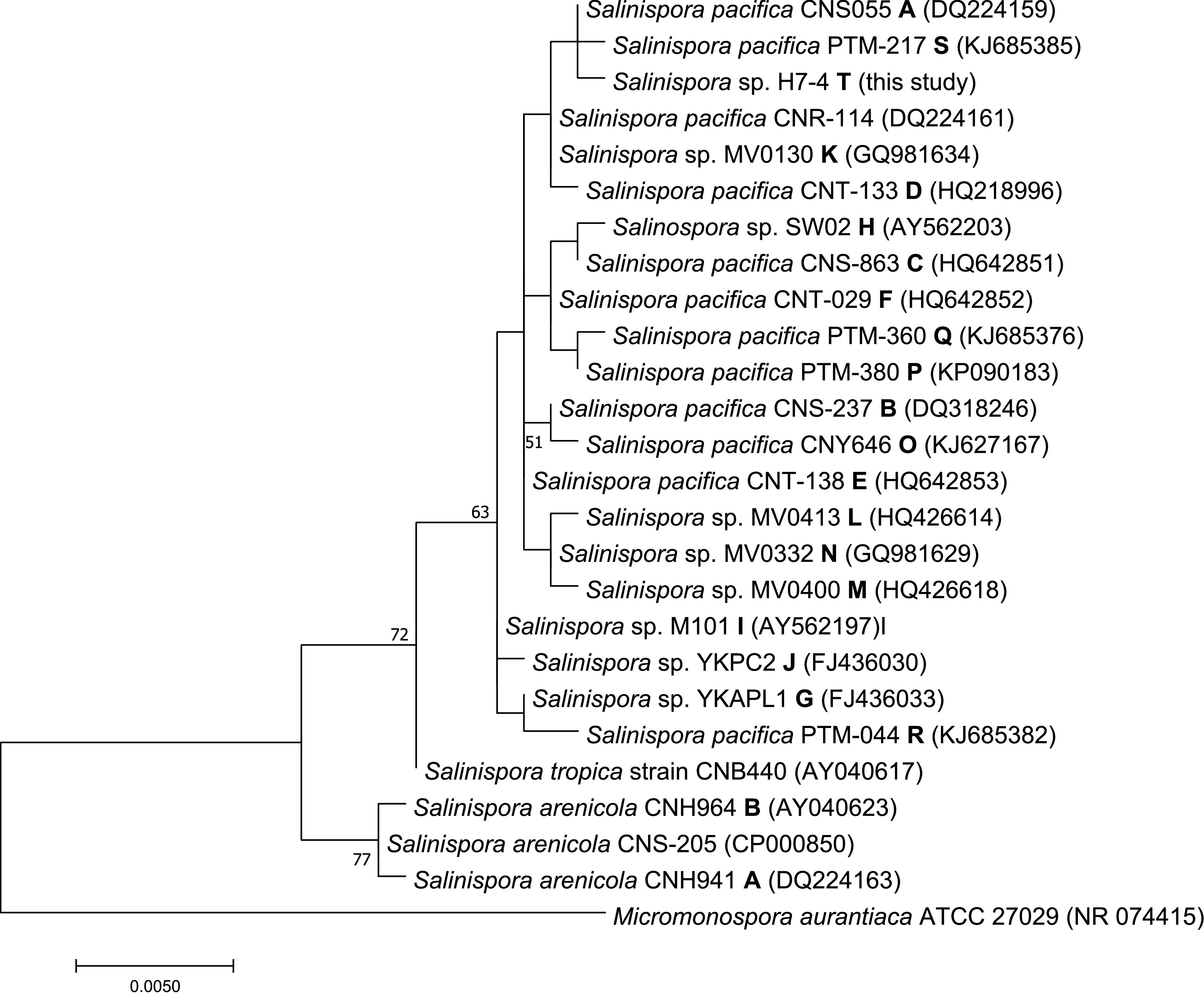
Maximum likelihood phylogenetic tree using 16S rRNA gene sequences (1,171 nucleotide positions) from representative strains of each *Salinispora* phylotype. Species names are followed by the strain identifier, 16S rRNA gene sequence phylotype (the type strain sequence is not labeled), and GenBank accession number in parentheses. Micromonospora aurantiaca ATCC 27029 was used as an outgroup. Bootstrap values of >50% are shown for 1,000 replicates at the respective nodes. The alignment and tree were constructed using MEGA software version 7.0.26 (13).

The seawater-dependent *Salinispora*-like colony H7-4 with an orange substrate mycelium was isolated from M1 agar. The 16S rRNA gene sequence BLASTn analysis (nucleotide collection database, BLASTn default settings) revealed that the isolate shared 99.93% identity with the best-hit *S. pacifica* strain CNS-055, indicating that strain H7-4 is a new *Salinispora* phylotype (T) in the *S. pacifica* clade (Fig. 1). For genomic DNA extraction, liquid AMM was inoculated with a mass of *Salinispora* sp. strain H7-4 mycelium and incubated for 10 days at 28°C at 160 rpm. The genomic DNA was extracted using the genomic DNA buffer set and Genomic-tip 20/G (both from Qiagen) according to the manufacturer’s protocol. The DNA quantity and quality were evaluated with a Qubit version 3.0 fluorometer (Thermo Fisher Scientific) and a NanoVue Plus spectrophotometer (GE Healthcare).

Library preparation and sequencing were performed at the Beijing Genomics Institute (BGI) (Hong Kong). The library was prepared with the BGI library construction protocol and BGI-developed kits. Briefly, 1 μg genomic DNA was randomly fragmented by a Covaris sonicator. DNA fragments were end repaired and A tailed. Illumina adapters were ligated to the 3′-adenylated DNA, and the resultant fragments were size selected by agarose gel electrophoresis. Adapter-ligated DNA fragments were enriched by PCR amplification and purified with the QIAquick gel extraction kit (Qiagen). The library quality and quantity were assessed with the Agilent Technologies 2100 Bioanalyzer and ABI StepOnePlus real-time PCR system. The library sequencing was performed using the HiSeq 4000 platform (2 × 150 paired-end reads; Illumina). A total of 7,977,738 reads were generated. Sequence adapters were trimmed after sequencing using the BGI protocol. The generated reads with low quality were filtered out with fastq_quality_filter in the FASTX-Toolkit (http://hannonlab.cshl.edu/fastx_toolkit) with the options -Q 33 -q 20 -p 80. The 7,586,610 reads obtained were assembled by the Platanus-B assembler version 1.1.0 (10). The genome annotation was generated by the NCBI Prokaryotic Genome Annotation Pipeline (PGAP) (11). BGC prediction was done by antiSMASH version 5.1.2 (12). All of the tools except for the FASTX-Toolkit were run with default parameters.

The draft genome of *Salinispora* sp. strain H7-4 has a total length of 5,258,402 bp, distributed in 81 contigs, with the largest contig being 692,820 bp long. The average coverage was approximately 200×; the *N*_50_ value for the assembly was 219,917 bp, and the GC content was 70.17%. The annotation of *Salinispora* sp. strain H7-4 identified 4,847 total genes, including 4,789 total coding DNA sequences, 51 tRNAs, and 1 each of the complete 5S, 16S, and 23S rRNAs. The antiSMASH pipeline predicted the presence of 23 full or partial BGCs, including those for production of polyketides, nonribosomal peptides, and terpenes.

This study adds a new site of genus *Salinispora* detection and extends the knowledge on the biogeography of these marine actinomycetes. These genomic data can be used for comparative genomic analyses, as well as BGC distribution and evolution studies.

### Data availability.

This whole-genome shotgun project has been deposited in DDBJ/ENA/GenBank under the accession number JABZED000000000. The version described in this paper is version JABZED010000000. The raw reads are available in the Sequence Read Archive (SRA) under BioProject accession number PRJNA641760.
